# Blood Pressure Reduction Time Windows in Stroke: A Systematic Review and Network Meta‐Analysis

**DOI:** 10.1002/brb3.71706

**Published:** 2026-08-24

**Authors:** Xinmin Yu, Chenlei Shi, Weiwei Wang, Degang Xue, Yuting Jiang, Miaomiao Zhao

**Affiliations:** ^1^ Jinshan Central Hospital Affiliated to Shanghai University of Medicine & Health Sciences Shanghai China; ^2^ School of Clinical Medicine Shanghai University of Medicine & Health Sciences Shanghai China; ^3^ Department of Public Health Jinshan Central Hospital Affiliated to Shanghai University of Medicine & Health Sciences Shanghai China; ^4^ Department of Cardiology General Hospital of Fushun Mining Bureau of Liaoning Health Industry Fushun Liaoning Province China

**Keywords:** blood pressure reduction, network meta‐analysis, stroke, time window

## Abstract

**Background:**

The optimal time windows for blood pressure reduction (BPR) treatment in patients with stroke remain controversial. This systematic review and network meta‐analysis (NMA) evaluates the efficacy of different BPR time windows across stroke subtypes and assesses associated safety outcomes.

**Methods:**

A systematic review of RCTs was conducted using PubMed, Embase, Cochrane Central Register of Controlled Trials, and Web of Science from their inception to March 21, 2026. Eligible trials enrolled patients with stroke treated with BPR. The primary outcome was mortality during follow‐up. Confidence in Network Meta‐Analysis (CINeMA) and the Cochrane Risk of Bias tool were used to assess quality. A random‐effects meta‐analysis model was employed to pool the odds ratios (ORs) and mean differences, with their corresponding 95% confidence intervals (CIs) reported.

**Results:**

A total of 31 randomized controlled trials (RCTs), including 29,276 patients, were included. For ischemic stroke (IS) patients (13 RCTs, *n* = 17,220), prehospital BPR therapy was associated with increased mortality risk compared with the reference (OR 1.28, 95% CI [1.01, 1.61]) and the 48 h window (OR 1.34, 95% CI [1.01, 1.77]). In intracerebral hemorrhage (ICH) patients (13 RCTs, *n* = 6826) and unspecified stroke patients (13 RCTs, n = 13,323), BPR therapy had no significant impact on mortality at any time window. Subgroup analyses were consistent with the primary findings.

**Conclusions:**

This NMA suggests a potential association between prehospital BPR treatment and increased risk of stroke mortality in IS patients versus the reference and within 48 h BPR groups, although evidence confidence is limited. These findings support cautious prehospital BPR use pending stroke subtype confirmation and highlight the need for further high‐quality studies.

## Graphical abstract

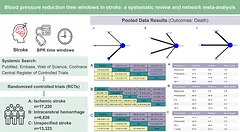

## 1 Introduction

Stroke is the second leading cause of mortality and the third leading cause of combined mortality and disability worldwide (GBD 2019 Diseases and Injuries Collaborators 2020; GBD 2019 Stroke Collaborators 2021; GBD 2021 Stroke Risk Factor Collaborators 2024). It is associated with unfavorable outcomes, including recurrent stroke, death, and permanent disability (Go et al. 2014). Over the past three decades, the total number of stroke‐related deaths or disabilities has nearly doubled, with recurrent stroke accounting for 25%‐30% of the overall stroke burden (Hankey 2014). The risk of mortality following recurrent stroke is significantly higher than that after a first‐time stroke. For instance, a Danish study revealed that the 1‐year and 10‐year all‐cause mortality rates after an initial ischemic stroke (IS) were 17% and 56%, respectively, whereas these rates increased to 25% and 70% after recurrence (Skajaa et al. 2022). The occurrence of secondary cardiovascular events after stroke poses a substantial threat to public health and imposes considerable burdens on healthcare resources globally.

Elevated blood pressure is the primary modifiable risk factor for secondary events after both IS and intracerebral hemorrhage (ICH) (GBD 2019 Stroke Collaborators 2021; Go et al. 2014; Willmot et al. 2004). Indeed, the incidence of recurrent stroke has decreased significantly over recent decades, largely due to advancements in blood pressure management (Hong et al. 2011). However, recent randomized controlled trials (RCTs) have reported inconsistent findings regarding the effect of blood pressure reduction (BPR) on secondary events after stroke. The INTERACT4 trial demonstrated that prehospital BPR therapy in IS patients was associated with an increased risk of poor functional outcomes (Li et al. 2024), while the MR ASAP and RIGHT2 trials reported neutral effects on functional outcomes (van den Berg et al. 2022; RIGHT‐2 Investigators 2019). In ICH patients, early BPR therapy initiated within 6 h reduced the risk of hematoma expansion and improved functional outcomes (Zhao et al. 2019); however, the ATACH‐2 trial showed no significant effect on functional recovery (Qureshi et al. 2016).

Several contemporary meta‐analyses of RCTs have further clarified the effects of BPR therapy across different time windows in stroke patients (Zaki et al. 2023; Yuan et al. 2025; Yu et al. 2023; Wang et al. 2014; Moullaali et al. 2022; Lee et al. 2015; Katsanos et al. 2017; Ibrahim et al. 2025; Gong et al. 2017). Nevertheless, the optimal time window for BPR therapy in different stroke types remains controversial. Therefore, to address this gap, we conducted a systematic review and network meta‐analysis (NMA) of all relevant RCTs to evaluate the impact of BPR interventions within different time windows on mortality and its subgroups in patients with IS, ICH, and unspecified stroke, respectively.

## 2 Materials and Methods

A systematic review and NMA of RCTs were conducted to investigate the association between BPR treatment time windows and clinical outcomes in stroke patients. This study is registered with PROSPERO (CRD420251022141) and reported in accordance with PRISMA 2020 guidelines.

### 2.1 Search Strategy and Selection Criteria

Articles evaluating BPR in stroke were independently reviewed by two reviewers. A second independent reviewer repeated the search to ensure the comprehensive inclusion of relevant articles. We searched the following databases from their inception until March 21 2026 without language restrictions: Embase, PubMed, Web of Science, and the Cochrane Central Register of Controlled Trials. The search strategy utilized the following terms:

((stroke) OR (acute stroke) OR (ischemic stroke) OR (intracranial hemorrhage) OR (cerebral hemorrhage) OR (hemorrhagic stroke). AND ((blood pressure reduction) OR (blood pressure) OR (blood pressure‐lowering))

Potentially relevant articles were reviewed in full text to ensure they met all the following criteria: (1) The article was an RCT; (2) All participants were 18 years old or older; (3) The intervention resulted in BPR; (4) The outcome evaluation time was not restricted.

### 2.2 Study Quality Assessment

Two independent reviewers assessed the methodological quality and risk of bias of each study using the Cochrane Risk of Bias tool (Higgins and Welton 2015) in the following seven domains: (1) random sequence generation, (2) allocation concealment, (3) blinding of participants and personnel, (4) blinding of outcome assessment, (5) incomplete outcome data, (6) selective reporting, and (7) other bias. Discrepancies between reviewers were resolved through discussion to reach consensus; if unresolved, a third reviewer was consulted, and their decision was deemed final. We performed the Confidence in Network Meta‐Analysis (CINeMA) to evaluate the certainty of evidence across six key dominants, including within‐study bias, reporting bias, indirectness, imprecision, heterogeneity, and inconsistency (Nikolakopoulou et al. 2020; Papakonstantinou et al. 2020).

### 2.3 Data Extraction

Duplicates were removed with EndNote 20, and two independent reviewers screened titles/abstracts and subsequent full texts of potentially eligible studies. Two reviewers independently extracted data on the name of the first author, publication year, participant demographics, sample size, and interventions from included trials, with cross‐validation by a third reviewer to ensure data accuracy. Baseline characteristics were tabulated. For the NMA, odds ratios (ORs) with 95% confidence intervals (CIs) served as the primary effect size measure. The Cochrane inverse variance method was used to derive unreported ORs, and variance‐adjusted approaches were applied for multi‐arm trials (Higgins et al. 2024).

### 2.4 Outcomes

The primary outcome was mortality in patients with IS, ICH, and unspecified stroke, with no restriction on the follow‐up time window.

### 2.5 Statistical Analysis

A frequentist NMA was performed using the “network” package in Stata MP 18.0. ORs with 95% CIs were calculated for dichotomous outcomes. Heterogeneity in each pairwise comparison was assessed using the *I* ^2^ statistic and *p*‐value (Higgins and Thompson 2002). Given potential heterogeneity across included studies, a random‐effects model was used to pool results. Global network consistency was assessed using node‐splitting analysis for comparisons with closed loops. For comparisons without closed loops, consistency could not be assessed; instead, we evaluated between‐study heterogeneity using the I^2^ statistic. An I^2^ value of 0–50% was considered low heterogeneity, 50–75% moderate heterogeneity, and 75–90% high heterogeneity.

Due to the star‐shaped structure of all three evidence networks, closed loops were absent, rendering formal node‐splitting consistency tests infeasible. Consequently, all comparisons between BPR time windows were derived indirectly, exclusively through the common reference node, with no direct evidence available to validate them.

According to established clinical nomenclature (Bath and Krishnan 2014; Allen et al. 2012), assignments to time window nodes were based on the upper bound of the reported treatment window and are detailed in Supplementary Table S1.

Transitivity constitutes the fundamental assumption for a valid NMA. Accordingly, transitivity was evaluated by comparing the distribution of potential effect modifiers (including age, course of disease, and sample size) across all treatment groups to verify the eligibility of included studies for NMA.

Rank probabilities for all BPR time windows were calculated, and the treatment hierarchy was presented as the surface under the cumulative ranking curve (SUCRA) (Salanti et al. 2011); lower SUCRA values indicate a greater probability of elevated mortality risk. League tables were constructed to illustrate relative treatment effects. Funnel plots were generated to assess publication bias (Higgins et al. 2024) when 10 or more studies were included.

Sensitivity analyses were conducted by (1) excluding articles with sample sizes of less than 30 in either the intervention or reference groups (Zhou and Shen 2022) and (2) sequentially removing trials of each antihypertensive drug or regimen (lisinopril, nimodipine, urapidil, candesartan, transdermal glyceryl trinitrate (GTN), magnesium sulfate, valsartan, prostacyclin (PGI_2_), and combinations of two or more drugs. All sensitivity analyses were repeated by stroke subtype.

Subgroup analyses were conducted to stratify outcomes by stroke subtype and, specifically, to test whether pooling different control types into a single reference node was justified. To this end, we performed predefined subgroup analyses stratified by control type (placebo, usual care, standard BPR treatment, and no BPR treatment) within each time window and stroke subtype.

## 3 Results

### 3.1 Study Selection and Characteristics

**FIGURE 1:**
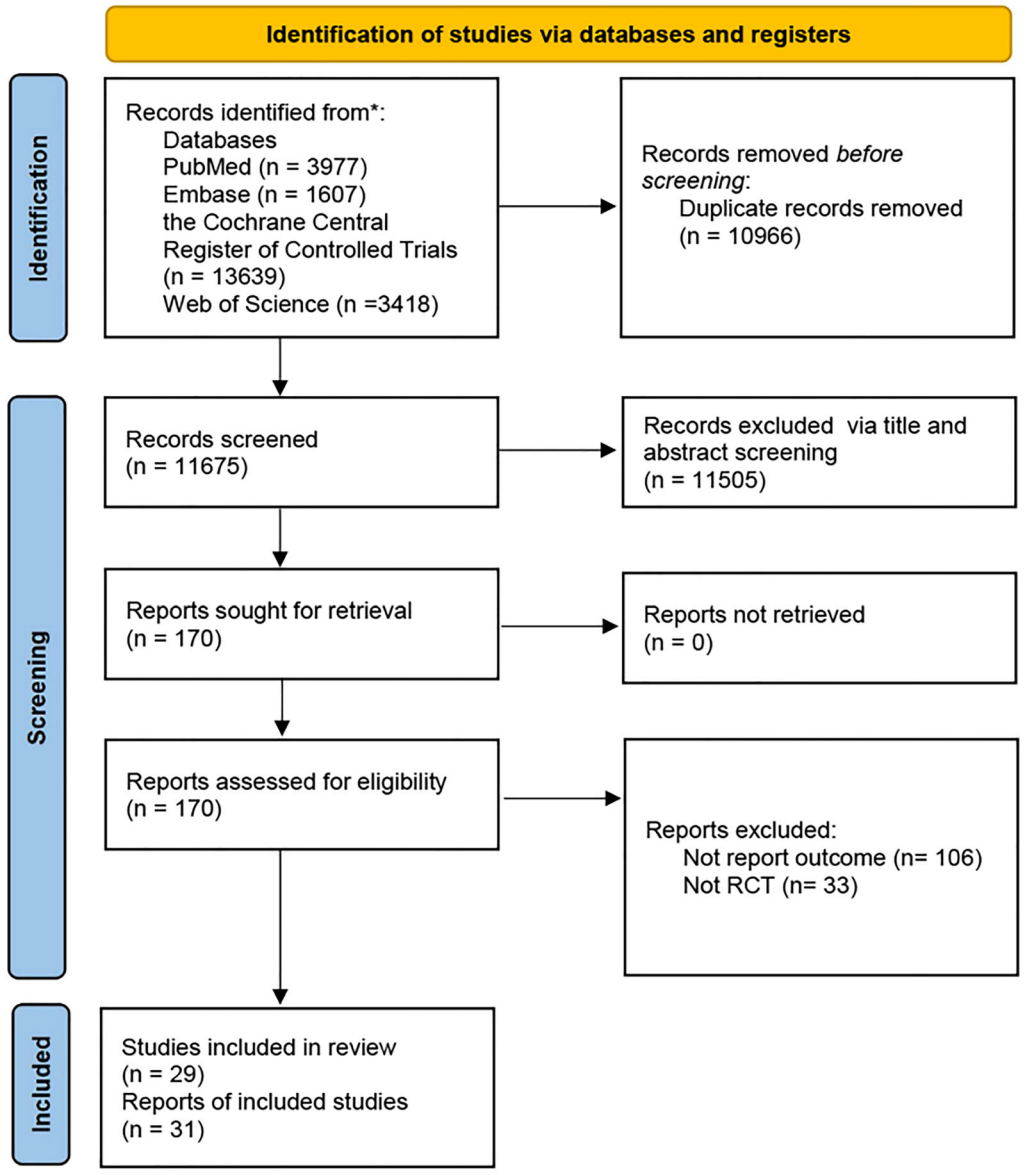
Selection of studies. The flowchart presents the stepwise progression of study selection, from the initial database search (*n* = 22,641) to the final inclusion of 31 reports in the network meta‐analysis. Intermediate counts are shown for duplicates removed (*n* = 10,966), records excluded during title/abstract screening (*n* = 11,505), and full‐text articles excluded (*n* = 139).

**FIGURE 2:**
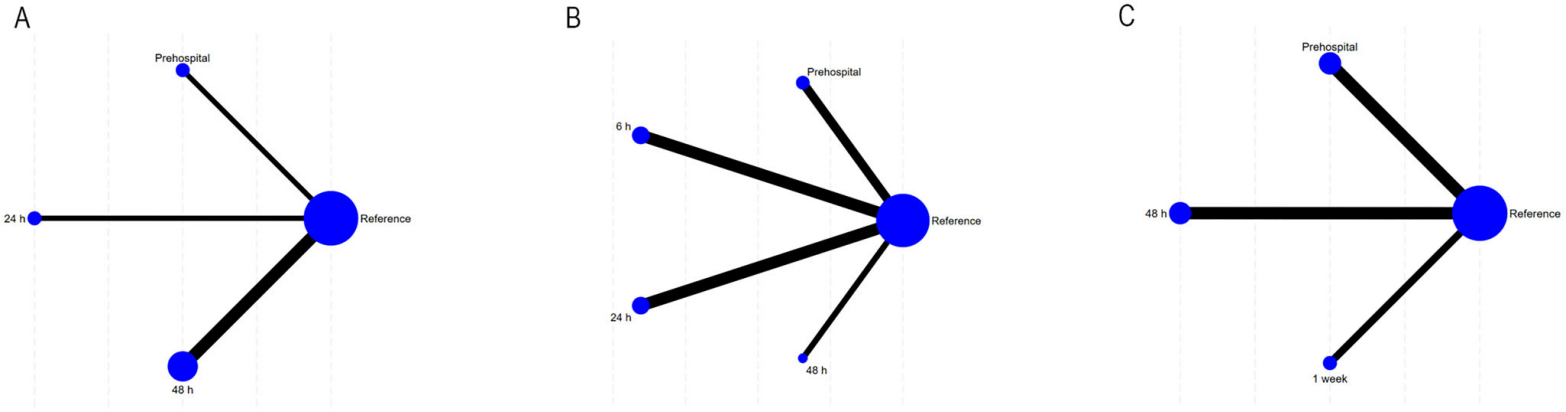
Network diagrams for (A) ischemic stroke, (B) intracerebral hemorrhage, and (C) unspecified stroke subtypes. Each node represents a blood pressure reduction intervention, with node size proportional to the total number of participants receiving that intervention. Connecting lines indicate direct comparisons between interventions based on the included randomized controlled trials (RCTs), and line thickness is proportional to the number of studies supporting each pairwise comparison. The network comprises the following six treatment nodes: reference, prehospital, 6 h, 24 h, 48 h, and 1 week blood pressure reduction.

Literature search initially identified 22,641 unique articles. Following title and abstract screening, 170 articles were selected for full‐text evaluation. Ultimately, 31 articles were included, corresponding to 29 unique RCTs, with two trials reported in two separate articles each (Figure 1). The key characteristics of the included studies are summarized in Table 1. The reference group was defined as receiving delayed BPR therapy, standard BPR therapy, placebo, or no BPR therapy. The evidence network for eligible comparisons of mortality outcomes is presented in Figure 2.

**TABLE 1:** Characteristics of included trials.

Author, Year	Population	Course of disease, h	Sample size (% men)	Age, y	Intervention	Control
(Li et al. 2024)	Acute stroke, SBP≥ 150 mm Hg	2	2404 (61.7)	70	Urapidil for 7 days	Usual blood‐pressure management for 7 d
(Ankolekar et al. 2013)	Acute stroke, SBP≥ 140 mm Hg	4	41 (53.7)	79	Transdermal GTN for 7 days	No transdermal GTN
(RIGHT‐2 Investigators 2019)	Acute stroke, SBP≥ 120 mm Hg	4	1149 (51.7)	74.5	Transdermal GTN for 4 days	No transdermal GTN
(van den Berg et al. 2022)	Acute stroke, SBP≥ 140 mm Hg	3	325 (52.6)	72	Transdermal GTN for 1 day	No transdermal GTN
(Saver et al. 2015)	Acute stroke	2	1700 (57.4)	69	Magnesium sulfate for 24 h	Placebo for 24 h
(Hsu et al. 1987)	Acute nonhemorrhagic stroke, SBP≤ 200 mm Hg	24	80 (61.3)	64	PGI_2_ for 72 h	Placebo for 72 h
(Eveson et al. 2007)	Acute ischemic stroke, SBP≥ 140 mm Hg	24	25 (62.5)	74	Lisinopril for 14 days	Placebo for 14 days
(Ahmed et al. 2000)	Acute ischemic stroke, SBP≥ 140 mm Hg	24	265 (NA)	NA	Nimodipine for 14 days	Placebo for 14 days
(Oh et al. 2015)	Acute ischemic stroke, SBP: 150–185 mm Hg	48	393 (59.0)	65	Valsartan for 7 days	Placebo for 7 days
(Kaste et al. 1994)	Acute ischemic hemispheric stroke	48	350 (67.1)	58	Nimodipine for 21 days	Placebo for 21 days
(Liu et al. 2023)	Acute ischemic stroke, SBP: 140–219 mm Hg	48	4802 (64.8)	64	Early antihypertensive treatment	Delayed antihypertensive treatment
(He et al. 2014)	Acute ischemic stroke, SBP: 140–219 mm Hg	48	4071 (64.0)	62	Antihypertensive treatment for 7 days	No antihypertensive treatment
(Martínez‐Vila et al. 1990)	Acute ischemic cerebral infarction, SBP≥ 100 mm Hg	48	123 (56.9)	72	Nimodipine for 28 days	Placebo for 28 days
(Anderson et al. 2013)	Acute intracerebral hemorrhage, SBP: 150–220 mm Hg	6	2839 (62.7)	64	Intensive blood‐pressure lowering for 7 days	Guideline recommended blood‐pressure lowering for 7days
(Antihypertensive Treatment of Acute Cerebral Hemorrhage (ATACH) Investigators. 2010)	Acute cerebral hemorrhage, SBP≥ 170 mm Hg	6	60 (56.7)	62	Nimodipine	Nimodipine
(Anderson et al. 2008)	Acute cerebral hemorrhage, SBP: 150–220 mm Hg	6	404 (64.9)	63	Intensive blood‐pressure lowering for 7 days	Guideline recommended blood‐pressure lowering for 7days
(Qureshi et al. 2016)	Acute cerebral hemorrhage, SBP< 180 mm Hg	4.5	1000 (62.0)	62	Intensive treatment for 7 days	Standard treatment for 7 days
(Koch et al. 2008)	Acute intracerebral hemorrhage, MAP≥ 110 mm Hg	8	42 (54.8)	61	Intensive treatment	Standard treatment
(Dong et al. 2024)	Spontaneous intracerebral hemorrhage, SBP≥ 150 mm Hg	24	338 (68.6)	58	Intensive blood‐pressure lowering for 7 days	Guideline recommended blood‐pressure lowering for 7days
(Qureshi et al. 2005)	Acute intracerebral hemorrhage, SBP≥ 160 mm Hg	24	35 (47.1)	65	Antihypertensive treatment	No antihypertensive treatment
(Butcher et al. 2013)	Acute intracerebral hemorrhage, SBP> 150 mm Hg	24	75 (72.0)	70	Intensive treatment	Standard treatment
(Jusufovic et al. 2014)	Acute hemorrhagic stroke, SBP ≥ 140 mm Hg	30	274 (67.9)	68	Candesartan for 7days	Placebo for 7 days
(Krishnan et al. 2016)	Acute intracerebral hemorrhage, SBP ≥ 140 mm Hg	48	629 (66.0)	67	Transdermal GTN for 7 days	No transdermal GTN
(Investigators 2015)	Acute stroke, SBP: 140–220 mm Hg	48	4011 (57.3)	70	Transdermal GTN for 7 days	No transdermal GTN
(Sandset et al. 2011)	Acute stroke, SBP≥ 140 mm Hg	30	2029 (58.0)	71	Candesartan for 7 days	Placebo for 7 days
(Schrader et al. 2003)	Stroke, SBP≥ 200 mm Hg	36	339 (30.1)	68	Candesartan for 7 days	Placebo for 7 days
(Potter et al. 2009)	Stroke, SBP> 160 mm Hg	36	172 (55.2)	74	Lisinopril or labetolol for 14 days	Placebo for 14 days
(Robinson et al. 2010)	Stroke, SBP≤ 200 mm Hg	48	763 (55.8)	74	Antihypertensive treatment for 14 days	No antihypertensive treatment for 14 days
(Willmot et al. 2006)	Stroke, SBP: 140–220 mm Hg	120	18 (27.8)	70	Transdermal GTN for 7 days	No transdermal GTN
(Bath et al. 2001)	Stroke	120	37 (48.6)	74	Transdermal GTN for 12 days	Placebo for 12 days
(Yuan et al. 2021)	Stroke, SBP: 150–210 mm Hg	72	483 (55.7)	67	Intensive treatment	Standard treatment

Abbreviations: GTN = glyceryl trinitrate, MAP = mean arterial blood pressure, PGI2 = prostacyclin, SBP = systolic blood pressure.

For comparisons without closed loops, local consistency was assessed through between‐study heterogeneity, which demonstrated low heterogeneity for mortality across different BPR time windows in IS, ICH, and unspecified stroke patients (IS: *I* ^2^ = 0.0%, *p* = 0.456; ICH: *I* ^2^ = 6.3%, *p* = 0.383; unspecified stroke: *I* ^2^ = 4.2%, *p* = 0.404).

### 3.2 Risk of Bias in Included Trials

**FIGURE 3:**
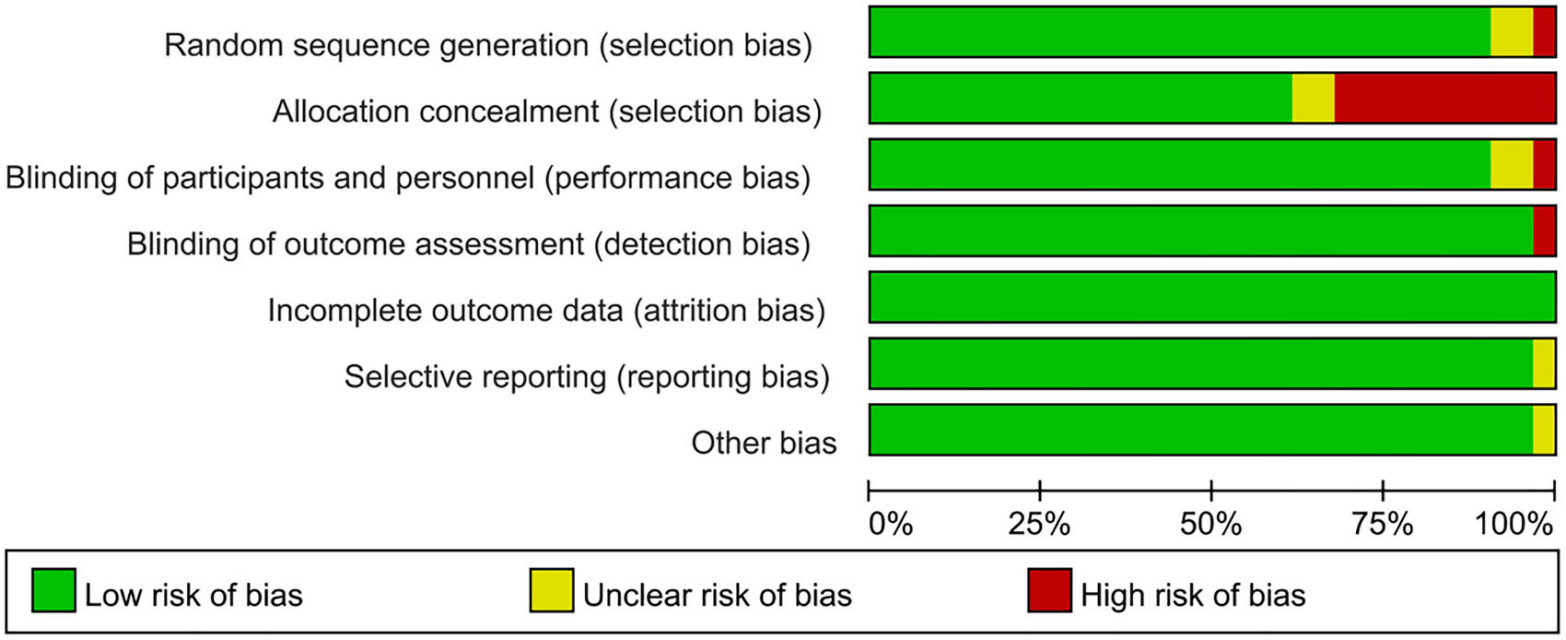
Summary of risk of bias assessment. Results are presented as percentages across the seven domains of the Cochrane Risk of Bias Tool, including random sequence generation, allocation concealment, blinding of participants and personnel, blinding of outcome assessment, incomplete outcome data, selective reporting, and other potential sources of bias. Bars represent the distribution of low (green), unclear (yellow), and high (red) risk of bias judgments for the included studies.

The risk of bias across included trials is summarized in Figure 3. Of the studies, 55% were categorized as having a low risk of bias, 13% had some concerns, and 32% were rated as having a high risk of bias. Funnel plots for each outcome are shown in Supplementary Figure S1. Egger's tests yielded no significant evidence of small‐study effects (Supplementary Table S2). The certainty of evidence for most pairwise comparisons was rated as low or very low confidence after assessing the level of evidence using CINeMA (Supplementary Tables S3–5).

To assess the transitivity assumption, we compared the distribution of key clinical variables, including age, course of disease, and sample size, across all treatment comparisons. No evidence of transitivity violation was found for the primary outcome across BPR time windows (Supplementary Figure S2). However, it was not feasible to detect potential intransitivity to a certain extent with the available data due to limited data availability and insufficient reporting of potential effect modifiers across included studies.

### 3.3 Ischemic Stroke

**FIGURE 4:**
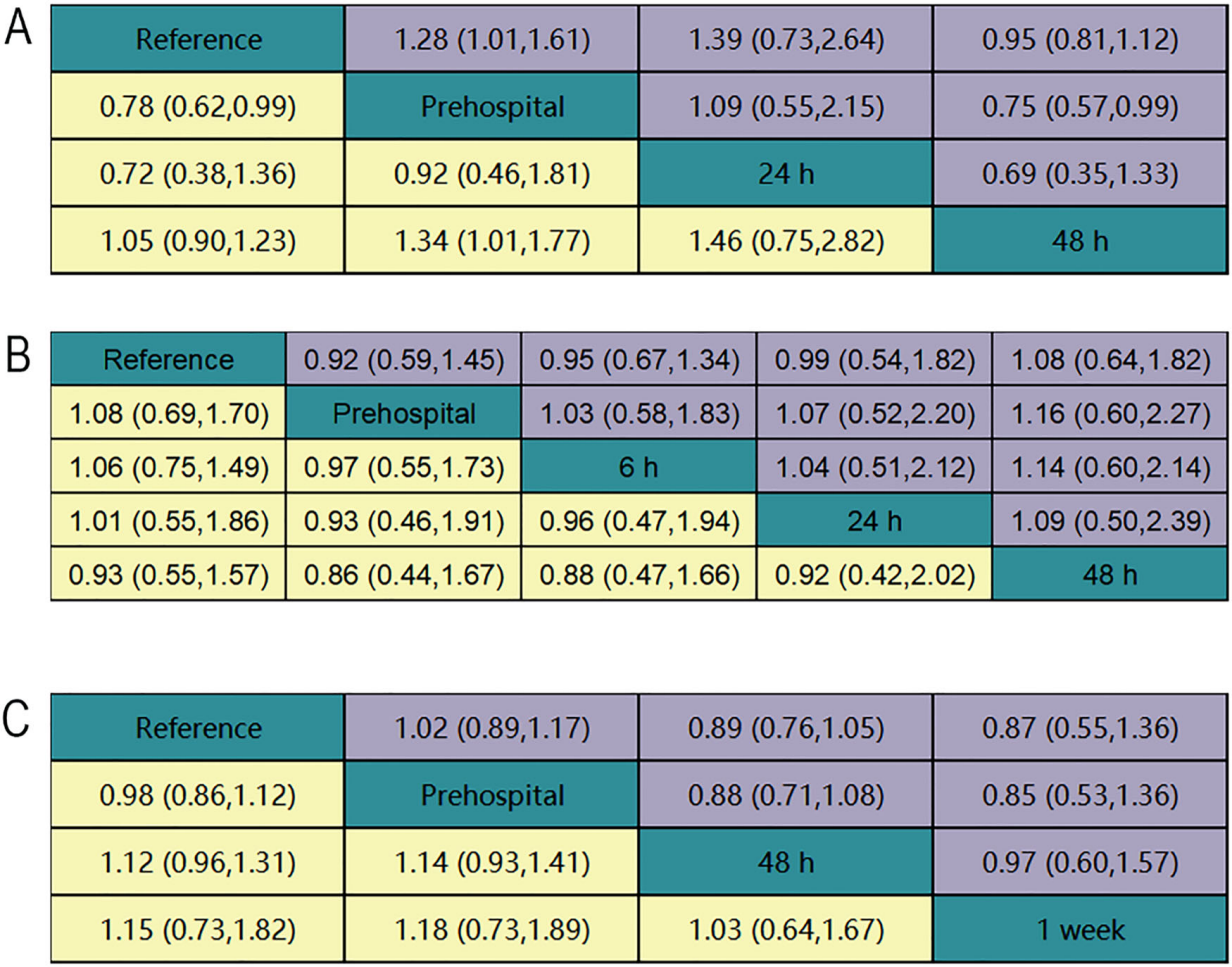
Network meta‐analysis of mortality for (A) ischemic stroke patients, (B) intracerebral hemorrhage patients, and (C) unspecified stroke patients, presented as pairwise comparisons between interventions. Data are odds ratios (ORs) with 95% confidence intervals (CIs) in a row‐to‐column format. ORs > 1 indicate that the column intervention is associated with a higher risk of mortality than the row intervention. Statistical significance is indicated when the 95% CI does not cross 1.

Thirteen RCTs involving 17,220 patients with IS were analyzed to evaluate BPR time windows, including prehospital, 24 h, and 48 h intervals. The results of the NMA for the outcomes are presented in Figure 4. Notably, only prehospital BPR was associated with increased mortality compared to the reference group (OR 1.28, 95% CI [1.01, 1.61]) and the 48 h window (OR 1.34, 95% CI [1.01, 1.77]).

**FIGURE 5:**
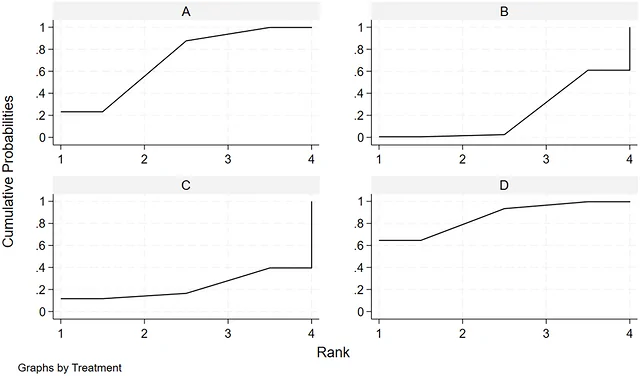
Surface under the cumulative ranking curve (SUCRA) for mortality in ischemic stroke patients across four interventions: (A) Reference, (B) Prehospital blood pressure reduction (BPR) treatment, (C) 24 h BPR treatment, and (D) 48 h BPR treatment. This plot displays the rank probabilities for each intervention, from most effective (rank 1, lowest mortality risk) to least effective (rank 4, highest mortality risk). Bar height represents the probability of achieving each specific rank. The SUCRA value reflects the probability that an intervention ranks among the best treatments, with higher values corresponding to more favorable average rankings.

For mortality, the 48 h window ranked highest by SUCRA (85.6%), followed by the reference group (70.2%), the 24 h window (22.8%), and prehospital BPR treatment (21.4%) (Figure 5 and Table 2).

**TABLE 2:** Rank of BPR treatment in ischemic stroke patients on mortality.

Treatment	SUCRA	PrBest	MeanRank
Reference	70.2	23.3	1.9
Prehospital	21.4	0.8	3.4
24 h	22.8	11.9	3.3
48 h	85.6	64.0	1.4

Abbreviations: BPR = blood pressure reduction, PrBest = probability of being best, SUCRA = surface under the cumulative ranking curve.

### 3.4 Intracerebral Hemorrhage

Thirteen RCTs, including 6826 patients diagnosed with ICH, were analyzed to assess BPR time windows, comprising prehospital, 6 h, 24 h, and 48 h intervals. For mortality outcomes, BPR interventions across all time windows showed no significant advantage compared to the reference group (Figure 4).

The SUCRAs for mortality indicate that the top three ranking interventions included prehospital BPR treatment (60.6%), followed by the 6 h window (57.0%) and the 24 h window (49.7%) (Supplementary Figure S3 and Table 3).

**TABLE 3:** Rank of BPR treatment in intracerebral hemorrhage patients on mortality.

Treatment	SUCRA	PrBest	MeanRank
Reference	45.7	4.6	3.2
Prehospital	60.6	30.8	2.6
6 h	57.0	23.5	2.7
24 h	49.7	26.9	3.0
48 h	36.9	14.2	3.5

Abbreviations: BPR = blood pressure reduction, PrBest = probability of being best, SUCRA = surface under the cumulative ranking curve.

### 3.5 Unspecified Stroke

Thirteen RCTs involving 13,323 patients presenting with unspecified strokes were included to analyze BPR time windows, including prehospital, 48 h, and 1 week intervals. Mortality outcomes showed no statistically significant difference between BPR and reference treatments across all time points (Figure 4).

The SUCRAs for mortality indicate that the top three ranking interventions included the 48 h window BPR treatment (75.3%), followed by the 1 week window (67.9%), and the reference group (31.8%) (Supplementary Figure S4 and Table 4).

**TABLE 4:** Rank of BPR treatment in unspecified stroke patients on mortality.

Treatment	SUCRA	PrBest	MeanRank
Reference	31.8	1.4	3.0
Prehospital	24.9	3.5	3.3
48 h	75.3	40.8	1.7
1 week	67.9	54.3	2.0

Abbreviations: BPR = blood pressure reduction, PrBest = probability of being best, SUCRA = surface under the cumulative ranking curve,.

### 3.6 Subgroup Analyses

In IS patients, neither 24 h nor 48 h BPR interventions demonstrated significant mortality reduction compared to the reference group. Similarly, among ICH patients, interventions initiated within 24 h or 48 h windows showed no statistically significant mortality improvement relative to the reference. For unspecified stroke patients, both 48 h and 1 week BPR protocols failed to achieve significant mortality reduction compared to controls. These results remained consistent with the primary analysis findings (Supplementary Figures S5–10).

In the subgroup analyses stratified by control type, no significant differences were detected among the reference group components for either ICH or unspecified stroke. For IS patients, prehospital BPR treatment remained associated with an increased risk of mortality, aligning with the findings of the primary analysis (Supplementary Figures S11–13).

### 3.7 Sensitivity Analyses

Sensitivity analyses demonstrated consistency with the primary outcome for IS (Supplementary Figures S14–15 and Supplementary Table S6). In patients with ICH or unspecified stroke, although the SUCRA rankings varied across BPR time windows, none of the treatments showed a statistically significant reduction in mortality compared with the reference group (Supplementary Figure S14, Supplementary Figures S16–17, and Supplementary Tables S7–8). Sensitivity analyses of antihypertensive drugs demonstrated that results were largely consistent with the primary outcome of IS, except when urapidil, candesartan, or transdermal GTN were sequentially excluded (Supplementary Figure S18). Results for ICH and unspecified stroke remained consistent with the primary outcome across all exclusions (Supplementary Figures S19–20).

## 4 Discussion

To our knowledge, this is the first NMA to compare BPR therapy time windows in patients with IS, ICH, and unspecified stroke. Previous studies have mostly emphasized the specific time window of BPR therapy in patients with a specific stroke subtype (Zaki et al. 2023; Wang et al. 2014; Lee et al. 2015). This analysis comprehensively evaluates mortality effects of diverse time windows for patients with IS, ICH, and unspecified stroke, comparing direct and indirect evidence. Additionally, to assess heterogeneity, we performed subgroup analyses of mortality at different follow‐up time points. Key findings in our study revealed that in patients with IS, prehospital BPR therapy was associated with increased risk of mortality compared to both the reference group and the group receiving BPR initiation within 48 h after stroke onset. Additionally, BPR therapy administered at any time window was associated with a neutral effect on mortality risk during follow‐up among patients with ICH or unspecified stroke.

For patients with IS, the SUCRA ranking from highest to lowest was 48 h window, reference group, 24 h window, and prehospital BPR treatment. Although the 48 h window ranked highest, there was no significant difference between the reference group and the 24 h windows, except for prehospital. Notably, prehospital BPR therapy may increase mortality risk in IS patients receiving BPR therapy within 48 h of onset or not receiving reperfusion therapies. These findings align with prior studies emphasizing the critical role of timing in BPR efficacy (Zaki et al. 2023; Yuan et al. 2025; Lee et al. 2015; Katsanos et al. 2017). Contrasting evidence emerged from a 2016 meta‐analysis focusing on intensive systolic blood pressure (SBP) reduction targets with time windows extending beyond 48 h, which reported reduced mortality with BPR (Katsanos et al. 2017). These discrepancies may arise from heterogeneity in treatment time windows, antihypertensive protocols, and baseline blood pressure levels. Over 60% of stroke patients exhibit acute hypertension upon emergency admission (Qureshi et al. 2007), yet this elevation often represents a physiologic compensatory response to ischemia and may resolve spontaneously without intervention (AlSibai and Qureshi 2016). Existing evidence indicates that focal cerebral hypoperfusion and stress responses accompanied by the activation of neuroendocrine systems are the primary etiological factors contributing to transient blood pressure elevation in acute ischemic stroke (Hadjiev and Mineva 2013). The dual role of elevated blood pressure in acute IS adds complexity to management. While it may preserve cerebral perfusion in penumbral regions, it also risks worsening cerebral edema or hemorrhagic transformation (McManus and Liebeskind 2016; Qureshi 2008). Current guidelines from the American Stroke Association (ASA) and European Stroke Initiative, therefore, recommend against routine BPR in acute IS unless SBP persistently exceeds 200–220 mmHg or diastolic blood pressure (DBP) >120 mmHg (Adams et al. 2007; Hacke et al. 2000), reflecting the lack of conclusive benefit and potential harm from aggressive early intervention.

However, prehospital BPR poses practical challenges. For instance, stroke type determination cannot rely solely on ambulance physicians’ clinical judgment. Simultaneously, prehospital BPR therapy may be harmful for patients with IS. Hence, given the challenges in prehospital diagnosis, recent advancements in mobile stroke units (MSUs), equipped with integrated CT scanners (Fassbender et al. 2003), have offered potential solutions to enhance the management of acute stroke, leading to better clinical outcomes and shorter treatment delays (Turc et al. 2022; Rohmann et al. 2023; Piccininni et al. 2023; Ebinger et al. 2021; Cooley et al. 2021). A multi‐center trial demonstrated that MSU treatment led to better functional outcomes for both AIS and ICH patients compared to traditional emergency medical services (EMS) (Grotta et al. 2021). Despite the promise shown by MSUs, challenges such as high costs and limited access to neurologists remain barriers to widespread implementation (Lund et al. 2022; Rink et al. 2024). To enhance MSU efficiency and mitigate constraints, integrating advanced diagnostic systems such as electroencephalography (EEG), transcranial Doppler (TCD), and Volumetric Impedance Phase Shift Spectroscopy (VIPS) headsets into prehospital care to facilitate rapid differentiation of ischemic and hemorrhagic stroke could be beneficial (Wolcott and English 2024).

In ICH patients, SUCRA values were highest for prehospital BPR treatment, followed by the 6 h, 24 h, reference, and 48 h windows. However, as none of the pairwise comparisons reached statistical significance, these rankings should be interpreted as descriptive only and do not reflect a true treatment hierarchy. Thus, the optimal time window for BPR therapy remains to be defined. Despite this uncertainty, the importance of BPR therapy in ICH is well recognized. Epidemiological data show a continued rise in hypertension‐related ICH (Tu and Wang 2023), and studies have confirmed that intensive antihypertensive therapy can effectively limit hematoma expansion (Zhao et al. 2019; Qureshi et al. 2016; Li et al. 2023). Recent evidence has shifted focus to the magnitude of blood pressure reduction. Divani et al. (2020) found that SBP reduction exceeding 40 mmHg within 6 h may be detrimental, a finding supported by the ATACH‐2 trial (Qureshi et al. 2016). Conversely, another study suggested an optimal reduction of 55–85 mmHg within 2 h for mild‐to‐moderate ICH (Li et al. 2025). These findings highlight the need for individualized blood pressure management in acute ICH, balancing hematoma control against the risk of cerebral hypoperfusion.

In unspecified strokes, SUCRA rankings placed the BPR treatment within 48 h and 1 week windows ahead of the reference group, but none of these comparisons reached statistical significance. These findings of neutral mortality effects across time windows align with 2014 studies on BPR for unspecified stroke (Bath and Krishnan 2014). This research enhances generalizability by incorporating recent RCTs such as INTERACT4 (Li et al. 2024), RIGHT‐2 (RIGHT‐2 Investigators 2019), CHASE (Yuan et al. 2021), et al. Moreover, we determined the ranking of the optimal time windows among various treatment scenarios, although there was no significant difference. Therefore, considering the outcomes of BPR therapy in IS and ICH patients, administering prehospital BPR without neuroimaging verification may potentially cause adverse effects on IS patients yet offer benefits to ICH patients, thereby blunting the overall efficacy of such treatment.

However, several limitations warrant acknowledgment. First, it is important to recognize that our evidence network has a star‐shaped structure, in which all interventions were compared only with a common control, with no direct head‐to‐head trials. Consequently, our NMA findings are derived solely from indirect comparisons. This sparse structure reflects the current state of the evidence base in this setting. Given this structural limitation of the network, the findings should be interpreted with due caution. Second, functional outcomes across stroke subtypes could not be compared owing to incomplete reporting in the original trials. Third, the transitivity assumption could not be rigorously evaluated due to insufficient reporting of key effect modifiers. Fourth, the methodological quality of the included studies was inconsistent. More than one‐third had a high risk of methodological bias, and the CINeMA assessment identified notable heterogeneity, imprecision, and incoherence, largely attributable to open‐label study designs and small sample sizes. Fifth, pooling trials enrolling unspecified stroke populations may have introduced clinical heterogeneity, given the distinct pathophysiological responses to blood pressure reduction in ischemic versus hemorrhagic stroke. Sixth, drug‐class sensitivity analyses were stable overall and in the ICH and unspecified stroke subgroups, but estimate changes occurred in the IS subgroup upon exclusion of one drug class, suggesting potential transitivity violations, possibly driven by drug‐specific cerebrovascular effects, the large sample size of the excluded studies, or both. Thus, findings for IS should be interpreted with caution.

## 5 Conclusion

In conclusion, available evidence suggests that prehospital BPR treatment may be associated with an increased risk of mortality in IS patients compared with the reference group and with those receiving BPR within 48 h, whereas no clear impact of BPR time windows on mortality was observed in ICH or unspecified stroke populations. There is an urgent need for additional multi‐center, double‐blind, placebo‐controlled trials to validate these findings and further investigate the optimal timing, safety, and efficacy of BPR therapy across IS, ICH, and unspecified stroke subtypes. Given the divergent pathological mechanisms of BPR treatment in ischemic versus hemorrhagic stroke, future studies should prioritize rapid subtype‐specific diagnosis to optimize the timing and targets of blood pressure management, ensuring personalized therapeutic strategies that align with stroke pathology.

## Author Contributions

**Yuting Jiang**: data curation. **Degang Xue**: data curation. **Xinmin Yu**: conceptualization, methodology, data curation, formal analysis, writing – original draft. **Chenlei Shi**: conceptualization, writing – original draft, methodology, formal analysis, data curation. **Miaomiao Zhao**: conceptualization, methodology, project administration, supervision, writing – review and editing. **Weiwei Wang**: data curation, formal analysis, writing – original draft.

## Funding

This study was funded by the National Natural Science Foundation of China (Grant No. 82003540), the Special Project for Clinical Research in the Health Industry of the Shanghai Municipal Health Commission (Grant No. 20194Y0116), and the Three‐Year Action Program of Shanghai Municipality for Strengthening the Construction of the Public Health System (Grant No. GWV10.2‐YQ39).

## Ethics Statement

The authors have no competing interests to declare that are relevant to the content of this article. This manuscript is a review article and does not involve a research protocol requiring approval by the relevant institutional review board or ethics committee.

## Conflicts of Interest

The authors declare no conflicts of interest.

## Supporting information




**Supporting information**: brb371706‐sup‐0001‐Figure1.tif

**Supporting information**: brb371706‐sup‐0002‐Figure2.tif

**Supporting information**: brb371706‐sup‐0003‐Figure3.tif

**Supporting information**: brb371706‐sup‐0004‐Figure4.tif

**Supporting information**: brb371706‐sup‐0005‐Figure5.tif

**Supporting information**: brb371706‐sup‐0006‐Figure6.tif

**Supporting information**: brb371706‐sup‐0007‐Figure7.tif

**Supporting information**: brb371706‐sup‐0008‐Figure8.tif

**Supporting information**: brb371706‐sup‐0009‐Figure9.tif

**Supporting information**: brb371706‐sup‐00010‐Figure10.tif

**Supporting information**: brb371706‐sup‐00011‐Figure11.tif

**Supporting information**: brb371706‐sup‐00012‐Figure12.tif

**Supporting information**: brb371706‐sup‐00013‐Figure13.tif

**Supporting information**: brb371706‐sup‐00014‐Figure14.tif

**Supporting information**: brb371706‐sup‐00015‐Figure15.tif

**Supporting information**: brb371706‐sup‐00016‐Figure16.tif

**Supporting information**: brb371706‐sup‐00017‐Figure17.tif

**Supporting information**: brb371706‐sup‐00018‐Figure18.tif

**Supporting information**: brb371706‐sup‐00019‐Figure19.tif

**Supporting information**: brb371706‐sup‐00020‐Figure20.tif

**Supplementary Figures**: brb371706‐sup‐00021‐SuppMat.docx

**Supplementary Tables**: brb371706‐sup‐00022‐SuppMat.docx

## Data Availability

The data supporting this study's findings are available from the corresponding author upon reasonable request.

## References

[brb371706-bib-0002] Adams, H. P. Jr. , G. del Zoppo , M. J. Alberts , et al. 2007. “Guidelines for the Early Management of Adults With Ischemic Stroke: Guideline From the American Heart Association/American Stroke Association Stroke Council, Clinical Cardiology Council, Cardiovascular Radiology and Intervention Council, and the Atherosclerotic Peripheral Vascular Disease and Quality of Care Outcomes in Research Interdisciplinary Working Groups: The American Academy of Neurology Affirms the Value of this Guideline as an Educational Tool for Neurologists.” Circulation 115: e478–534. 17515473 10.1161/CIRCULATIONAHA.107.181486

[brb371706-bib-0003] Ahmed, N. , P. Näsman , and N. G. Wahlgren . 2000. “Effect of Intravenous Nimodipine on Blood Pressure and Outcome After Acute Stroke.” Stroke; A Journal of Cerebral Circulation 31: 1250–1255. 10.1161/01.str.31.6.1250 10835440

[brb371706-bib-0004] Allen, L. M. , A. N. Hasso , J. Handwerker , and H. Farid . 2012. “Sequence‐Specific MR Imaging Findings That Are Useful in Dating Ischemic Stroke.” Radiographics 32: 1285–1297. 22977018 10.1148/rg.325115760

[brb371706-bib-0005] AlSibai, A. , and A. I. Qureshi . 2016. “Management of Acute Hypertensive Response in Patients with Ischemic Stroke.” The Neurohospitalist 6: 122–129. 27366297 10.1177/1941874416630029 PMC4906555

[brb371706-bib-0006] Anderson, C. S. , E. Heeley , Y. Huang , et al. 2013. “Rapid Blood‐Pressure Lowering in Patients With Acute Intracerebral Hemorrhage.” New England Journal of Medicine 368: 2355–2365. 23713578 10.1056/NEJMoa1214609

[brb371706-bib-0007] Anderson, C. S. , Y. Huang , J. G. Wang , et al. 2008. “Intensive Blood Pressure Reduction in Acute Cerebral Haemorrhage Trial (INTERACT): A Randomised Pilot Trial.” Lancet Neurology 7: 391–399. 18396107 10.1016/S1474-4422(08)70069-3

[brb371706-bib-0008] Ankolekar, S. , M. Fuller , I. Cross , et al. 2013. “Feasibility of an Ambulance‐based Stroke Trial, and Safety of Glyceryl Trinitrate in Ultra‐Acute Stroke: The Rapid Intervention With Glyceryl Trinitrate in Hypertensive Stroke Trial (RIGHT, ISRCTN66434824).” Stroke; A Journal of Cerebral Circulation 44: 3120–3128. 10.1161/STROKEAHA.113.001301 24003041

[brb371706-bib-0009] Bath, P. M. , and K. Krishnan . 2014. “Interventions for Deliberately Altering Blood Pressure in Acute Stroke.” Cochrane Database of Systematic Reviews 2014: Cd000039. 25353321 10.1002/14651858.CD000039.pub3 PMC7052738

[brb371706-bib-0010] Bath, P. M. , R. Pathansali , R. Iddenden , and F. J. Bath . 2001. “The Effect of Transdermal Glyceryl Trinitrate, a Nitric Oxide Donor, on Blood Pressure and Platelet Function in Acute Stroke.” Cerebrovascular Diseases 11: 265–272. 11306778 10.1159/000047649

[brb371706-bib-0011] Butcher, K. S. , T. Jeerakathil , M. Hill , et al. 2013. “The Intracerebral Hemorrhage Acutely Decreasing Arterial Pressure Trial.” Stroke; A Journal of Cerebral Circulation 44: 620–626. 10.1161/STROKEAHA.111.000188 23391776

[brb371706-bib-0012] Cooley, S. R. , H. Zhao , B. C. V. Campbell , et al. 2021. “Mobile Stroke Units Facilitate Prehospital Management of Intracerebral Hemorrhage.” Stroke; A Journal of Cerebral Circulation 52: 3163–3166. 10.1161/STROKEAHA.121.034592 34187178

[brb371706-bib-0013] Divani, A. A. , X. Liu , A. Petersen , et al. 2020. “The Magnitude of Blood Pressure Reduction Predicts Poor in‐Hospital Outcome in Acute Intracerebral Hemorrhage.” Neurocritica Care 33: 389–398. 10.1007/s12028-020-01016-z 32524527

[brb371706-bib-0014] Dong, R. , F. Li , B. Li , et al. 2024. “Effects of an Early Intensive Blood Pressure‐Lowering Strategy Using Remifentanil and Dexmedetomidine in Patients With Spontaneous Intracerebral Hemorrhage: A Multicenter, Prospective, Superiority, Randomized Controlled Trial.” Anesthesiology 141: 100–115. 38537025 10.1097/ALN.0000000000004986

[brb371706-bib-0015] Ebinger, M. , B. Siegerink , A. Kunz , et al. 2021. “Association Between Dispatch of Mobile Stroke Units and Functional Outcomes Among Patients With Acute Ischemic Stroke in Berlin.” JAMA 325: 454–466. 33528537 10.1001/jama.2020.26345 PMC7856548

[brb371706-bib-0016] Eveson, D. J. , T. G. Robinson , and J. F. Potter . 2007. “Lisinopril for the Treatment of Hypertension Within the First 24 Hours of Acute Ischemic Stroke and Follow‐up.” American Journal of Hypertension 20: 270–277. 17324738 10.1016/j.amjhyper.2006.08.005

[brb371706-bib-0017] Fassbender, K. , S. Walter , Y. Liu , et al. 2003. “‘Mobile Stroke Unit’ for Hyperacute Stroke Treatment.” Stroke; A Journal of Cerebral Circulation 34: e44. 10.1161/01.STR.0000075573.22885.3B 12750527

[brb371706-bib-0018] GBD 2019 Diseases and Injuries Collaborators . 2020. “Global Burden of 369 Diseases and Injuries in 204 Countries and Territories, 1990–2019: A Systematic Analysis for the Global Burden of Disease Study 2019.” Lancet 396: 1204–1222. 33069326 10.1016/S0140-6736(20)30925-9 PMC7567026

[brb371706-bib-0019] GBD 2019 Stroke Collaborators . 2021. “Global, Regional, and National Burden of Stroke and Its Risk Factors, 1990–2019: A Systematic Analysis for the Global Burden of Disease Study 2019.” Lancet Neurology 20: 795–820. 34487721 10.1016/S1474-4422(21)00252-0 PMC8443449

[brb371706-bib-0020] GBD 2021 Stroke Risk Factor Collaborators . 2024. “Global, Regional, and National Burden of Stroke and Its Risk Factors, 1990–2021: A Systematic Analysis for the Global Burden of Disease Study 2021.” Lancet Neurology 23: 973–1003. 39304265 10.1016/S1474-4422(24)00369-7 PMC12254192

[brb371706-bib-0021] Go, A. S. , D. Mozaffarian , V. L. Roger , et al. 2014. “Heart Disease and Stroke Statistics–2014 Update: A Report From the American Heart Association.” Circulation 129: e28–e292. 24352519 10.1161/01.cir.0000441139.02102.80 PMC5408159

[brb371706-bib-0022] Gong, S. , C. Lin , D. Zhang , et al. 2017. “Effects of Intensive Blood Pressure Reduction on Acute Intracerebral Hemorrhage: A Systematic Review and Meta‐Analysis.” Scientific Reports 7: 10694. 28878305 10.1038/s41598-017-10892-z PMC5587814

[brb371706-bib-0023] Grotta, J. C. , J. M. Yamal , S. A. Parker , et al. 2021. “Prospective, Multicenter, Controlled Trial of Mobile Stroke Units.” New England Journal of Medicine 385: 971–981. 34496173 10.1056/NEJMoa2103879

[brb371706-bib-0024] Hacke, W. , M. Kaste , T. Skyhoj Olsen , J. Bogousslavsky , and J. M. Orgogozo . 2000. “Acute Treatment of Ischemic Stroke. European Stroke Initiative (EUSI).” Cerebrovascular Diseases 10, no. 3: 22–33. 10940667 10.1159/000047578

[brb371706-bib-0025] Hadjiev, D. I. , and P. P. Mineva . 2013. “Elevated Blood Pressure Management in Acute Ischemic Stroke Remains Controversial: Could this Issue be Resolved?” Medical Hypotheses 80: 50–52. 23137749 10.1016/j.mehy.2012.10.008

[brb371706-bib-0026] Hankey, G. J. 2014. “Secondary Stroke Prevention.” Lancet Neurology 13: 178–194. 24361114 10.1016/S1474-4422(13)70255-2

[brb371706-bib-0027] He, J. , Y. Zhang , T. Xu , et al. 2014. “Effects of Immediate Blood Pressure Reduction on Death and Major Disability in Patients With Acute Ischemic Stroke: The CATIS Randomized Clinical Trial.” JAMA 311: 479–489. 24240777 10.1001/jama.2013.282543

[brb371706-bib-0028] Higgins, J. P. , and S. G. Thompson . 2002. “Quantifying Heterogeneity in a Meta‐Analysis.” Statistics in Medicine 21: 1539–1558. 12111919 10.1002/sim.1186

[brb371706-bib-0029] Higgins, J. P. , and N. J. Welton . 2015. “Network Meta‐Analysis: A Norm for Comparative Effectiveness?” Lancet 386: 628–630. 26334141 10.1016/S0140-6736(15)61478-7

[brb371706-bib-0030] Higgins, J. P. T. T. J. , J. Chandler , M. Cumpston , T. Li , M. J. Page , and V. A. Welch . *Cochrane Handbook for Systematic Reviews of Interventions* (Version 6.5 updated August 2024). Cochrane.

[brb371706-bib-0031] Hong, K. S. , S. Yegiaian , M. Lee , J. Lee , and J. L. Saver . 2011. “Declining Stroke and Vascular Event Recurrence Rates in Secondary Prevention Trials Over the Past 50 Years and Consequences for Current Trial Design.” Circulation 123: 2111–2119. 21536995 10.1161/CIRCULATIONAHA.109.934786 PMC3118516

[brb371706-bib-0032] Hsu, C. Y. , R. E. Faught Jr. , A. J. Furlan , et al. 1987. “Intravenous Prostacyclin in Acute Nonhemorrhagic Stroke: A Placebo‐Controlled Double‐Blind Trial.” Stroke; A Journal of Cerebral Circulation 18: 352–358. 10.1161/01.str.18.2.352 3551212

[brb371706-bib-0033] Ibrahim, A. A. , Y. Khlidj , A. M. Amin , et al. 2025. “Pre‐Hospital Blood Pressure Lowering in Presumed Hyperacute Stroke: A Systematic Review and Meta‐Analysis of Randomized Controlled Trials.” Journal of Stroke and Cerebrovascular Diseases 34: 108158. 39615639 10.1016/j.jstrokecerebrovasdis.2024.108158

[brb371706-bib-0034] Investigators, E. T. 2015. “Efficacy of Nitric Oxide, With or Without Continuing Antihypertensive Treatment, for Management of High Blood Pressure in Acute Stroke (ENOS): A Partial‐Factorial Randomised Controlled Trial.” Lancet 385: 617–628. 25465108 10.1016/S0140-6736(14)61121-1 PMC4343308

[brb371706-bib-0035] Jusufovic, M. , E. C. Sandset , P. M. Bath , and E. Berge . 2014. “Blood Pressure‐Lowering Treatment With Candesartan in Patients With Acute Hemorrhagic Stroke.” Stroke; A Journal of Cerebral Circulation 45: 3440–3442. 10.1161/STROKEAHA.114.006433 25256183

[brb371706-bib-0036] Kaste, M. , R. Fogelholm , T. Erilä , et al. 1994. “A Randomized, Double‐Blind, Placebo‐controlled Trial of Nimodipine in Acute Ischemic Hemispheric Stroke.” Stroke; A Journal of Cerebral Circulation 25: 1348–1353. 10.1161/01.str.25.7.1348 8023348

[brb371706-bib-0037] Katsanos, A. H. , A. Filippatou , E. Manios , et al. 2017. “Blood Pressure Reduction and Secondary Stroke Prevention: A Systematic Review and Metaregression Analysis of Randomized Clinical Trials.” Hypertension 69: 171–179. 27802419 10.1161/HYPERTENSIONAHA.116.08485

[brb371706-bib-0038] Koch, S. , J. G. Romano , A. M. Forteza , C. M. Otero , and A. A. Rabinstein . 2008. “Rapid Blood Pressure Reduction in Acute Intracerebral Hemorrhage: Feasibility and Safety.” Neurocritical Care 8: 316–321. 18360781 10.1007/s12028-008-9085-8

[brb371706-bib-0039] Krishnan, K. , P. Scutt , L. Woodhouse , et al. 2016. “Glyceryl Trinitrate for Acute Intracerebral Hemorrhage: Results From the Efficacy of Nitric Oxide in Stroke (ENOS) Trial, a Subgroup Analysis.” Stroke; A Journal of Cerebral Circulation 47: 44–52. 10.1161/STROKEAHA.115.010368 26645254

[brb371706-bib-0040] Lee, M. , B. Ovbiagele , K. S. Hong , et al. 2015. “Effect of Blood Pressure Lowering in Early Ischemic Stroke: Meta‐Analysis.” Stroke; A Journal of Cerebral Circulation 46: 1883–1889. 10.1161/STROKEAHA.115.009552 26022636

[brb371706-bib-0041] Li, G. , Y. Lin , J. Yang , et al. 2024. “Intensive Ambulance‐Delivered Blood‐Pressure Reduction in Hyperacute Stroke.” New England Journal of Medicine 390: 1862–1872. 38752650 10.1056/NEJMoa2314741

[brb371706-bib-0042] Li, Q. , X. Lv , A. Morotti , et al. 2025. “Optimal Magnitude of Blood Pressure Reduction and Hematoma Growth and Functional Outcomes in Intracerebral Hemorrhage.” Neurology 104: e213412. 39913881 10.1212/WNL.0000000000213412 PMC11803522

[brb371706-bib-0043] Li, Q. , A. Morotti , A. Warren , et al. 2023. “Intensive Blood Pressure Reduction Is Associated With Reduced Hematoma Growth in Fast Bleeding Intracerebral Hemorrhage.” Annals of Neurology 10, no. 7: 745–751. 10.1002/ana.26795 37706569

[brb371706-bib-0044] Liu, L. , X. Xie , Y. Pan , et al. 2023. “Early versus Delayed Antihypertensive Treatment in Patients With Acute Ischaemic Stroke: Multicentre, Open Label, Randomised, Controlled Trial.” Bmj 383: e076448. 37813418 10.1136/bmj-2023-076448 PMC10561001

[brb371706-bib-0045] Lund, U. H. , A. Stoinska‐Schneider , K. Larsen , K. G. Bache , and B. Robberstad . 2022. “Cost‐Effectiveness of Mobile Stroke Unit Care in Norway.” Stroke; A Journal of Cerebral Circulation 53: 3173–3181. 10.1161/STROKEAHA.121.037491 PMC9508956 35862205

[brb371706-bib-0046] Martínez‐Vila, E. , F. Guillén , J. A. Villanueva , et al. 1990. “Placebo‐Controlled Trial of Nimodipine in the Treatment of Acute Ischemic Cerebral Infarction.” Stroke; A Journal of Cerebral Circulation 21: 1023–1028. 10.1161/01.str.21.7.1023 2195714

[brb371706-bib-0047] McManus, M. , and D. S. Liebeskind . 2016. “Blood Pressure in Acute Ischemic Stroke.” Journal of Clinical Neurology 12: 6. 10.3988/jcn.2016.12.2.137 PMC4828558 26833984

[brb371706-bib-0048] Moullaali, T. J. , X. Wang , E. C. Sandset , et al. 2022. “Early Lowering of Blood Pressure After Acute Intracerebral Haemorrhage: A Systematic Review and Meta‐Analysis of Individual Patient Data.” Journal of Neurology, Neurosurgery, and Psychiatry 93: 6–13. 34732465 10.1136/jnnp-2021-327195 PMC8685661

[brb371706-bib-0049] Nikolakopoulou, A. , J. P. T. Higgins , T. Papakonstantinou , et al. 2020. “CINeMA: An Approach for Assessing Confidence in the Results of a Network Meta‐Analysis.” Plos Medicine 17: e1003082. 32243458 10.1371/journal.pmed.1003082 PMC7122720

[brb371706-bib-0050] Oh, M. S. , K. H. Yu , K. S. Hong , et al. 2015. “Modest Blood Pressure Reduction With Valsartan in Acute Ischemic Stroke: A Prospective, Randomized, Open‐Label, Blinded‐end‐point Trial.” International Journal of Stroke 10: 745–751. 25580869 10.1111/ijs.12446

[brb371706-bib-0051] Papakonstantinou, T. , A. Nikolakopoulou , J. P. T. Higgins , M. Egger , and G. Salanti . 2020. “CINeMA: Software for Semiautomated Assessment of the Confidence in the Results of Network Meta‐Analysis.” Campbell Systematic Reviews 16: e1080. 37131978 10.1002/cl2.1080 PMC8356302

[brb371706-bib-0052] Piccininni, M. , T. Kurth , H. J. Audebert , and J. L. Rohmann . 2023. “The Effect of Mobile Stroke Unit Care on Functional Outcomes: An Application of the Front‐Door Formula.” Epidemiology 34: 712–720. 37462471 10.1097/EDE.0000000000001642

[brb371706-bib-0053] Potter, J. F. , T. G. Robinson , G. A. Ford , et al. 2009. “Controlling Hypertension and Hypotension Immediately Post‐Stroke (CHHIPS): A Randomised, Placebo‐controlled, Double‐Blind Pilot Trial.” Lancet Neurology 8: 48–56. 19058760 10.1016/S1474-4422(08)70263-1

[brb371706-bib-0054] Qureshi, A. I. 2008. “Acute Hypertensive Response in Patients With Stroke: Pathophysiology and Management.” Circulation 118: 176–187. 18606927 10.1161/CIRCULATIONAHA.107.723874

[brb371706-bib-0055] Qureshi, A. I. , M. A. Ezzeddine , A. Nasar , et al. 2007. “Prevalence of Elevated Blood Pressure in 563,704 Adult Patients With Stroke Presenting to the ED in the United States.” American Journal of Emergency Medicine 25: 32–38. 17157679 10.1016/j.ajem.2006.07.008 PMC2443694

[brb371706-bib-0056] Qureshi, A. I. , Y. M. Mohammad , A. M. Yahia , et al. 2005. “A Prospective Multicenter Study to Evaluate the Feasibility and Safety of Aggressive Antihypertensive Treatment in Patients With Acute Intracerebral Hemorrhage.” Journal of Intensive Care Medicine 20: 34–42. 15665258 10.1177/0885066604271619

[brb371706-bib-0057] Qureshi, A. I. , Y. Y. Palesch , W. G. Barsan , et al. 2016. “Intensive Blood‐Pressure Lowering in Patients With Acute Cerebral Hemorrhage.” New England Journal of Medicine 375: 1033–1043. 27276234 10.1056/NEJMoa1603460 PMC5345109

[brb371706-bib-0001] Antihypertensive Treatment of Acute Cerebral Hemorrhage (ATACH) Investigators . “Antihypertensive Treatment of Acute Cerebral Hemorrhage.” Critical Care Medicine 38: 637–648. 19770736 10.1097/CCM.0b013e3181b9e1a5 PMC5568798

[brb371706-bib-0058] RIGHT‐2 Investigators . 2019. “Prehospital Transdermal Glyceryl Trinitrate in Patients With Ultra‐Acute Presumed Stroke (RIGHT‐2): An Ambulance‐Based, Randomised, Sham‐Controlled, Blinded, Phase 3 Trial.” Lancet 393: 1009–1020. 30738649 10.1016/S0140-6736(19)30194-1 PMC6497986

[brb371706-bib-0059] Rink, J. S. , F. Tollens , A. Tschalzev , et al. 2024. “Establishing an MSU Service in a Medium‐Sized German Urban Area‐Clinical and Economic Considerations.” Frontiers in Neurology 15: 1358145. 38487327 10.3389/fneur.2024.1358145 PMC10938346

[brb371706-bib-0060] Robinson, T. G. , J. F. Potter , G. A. Ford , et al. 2010. “Effects of Antihypertensive Treatment After Acute Stroke in the Continue or Stop Post‐Stroke Antihypertensives Collaborative Study (COSSACS): A Prospective, Randomised, Open, Blinded‐Endpoint Trial.” Lancet Neurology 9: 767–775. 20621562 10.1016/S1474-4422(10)70163-0

[brb371706-bib-0061] Rohmann, J. L. , M. Piccininni , M. Ebinger , et al. 2023. “Effect of Mobile Stroke Unit Dispatch in all Patients With Acute Stroke or TIA.” Annals of Neurology 93: 50–63. 36309933 10.1002/ana.26541

[brb371706-bib-0062] Salanti, G. , A. E. Ades , and J. P. Ioannidis . 2011. “Graphical Methods and Numerical Summaries for Presenting Results From Multiple‐Treatment Meta‐Analysis: An Overview and Tutorial.” Journal of Clinical Epidemiology 64: 163–171. 20688472 10.1016/j.jclinepi.2010.03.016

[brb371706-bib-0063] Sandset, E. C. , P. M. Bath , G. Boysen , et al. 2011. “The Angiotensin‐Receptor Blocker Candesartan for Treatment of Acute Stroke (SCAST): A Randomised, Placebo‐Controlled, Double‐Blind Trial.” Lancet 377: 741–750. 21316752 10.1016/S0140-6736(11)60104-9

[brb371706-bib-0064] Saver, J. L. , S. Starkman , M. Eckstein , et al. 2015. “Prehospital Use of Magnesium Sulfate as Neuroprotection in Acute Stroke.” New England Journal of Medicine 372: 528–536. 25651247 10.1056/NEJMoa1408827 PMC4920545

[brb371706-bib-0065] Schrader, J. , S. Lüders , A. Kulschewski , et al. 2003. “The ACCESS Study: Evaluation of Acute Candesartan Cilexetil Therapy in Stroke Survivors.” Stroke; A Journal of Cerebral Circulation 34: 1699–1703. 10.1161/01.STR.0000075777.18006.89 12817109

[brb371706-bib-0066] Skajaa, N. , K. Adelborg , E. Horváth‐Puhó , et al. 2022. “Risks of Stroke Recurrence and Mortality After First and Recurrent Strokes in Denmark: A Nationwide Registry Study.” Neurology 98: e329–e342. 34845054 10.1212/WNL.0000000000013118

[brb371706-bib-0067] Tu, W. J. , and L. D. Wang . 2023. “China Stroke Surveillance Report 2021.” Military Medical Research 10: 33. 37468952 10.1186/s40779-023-00463-x PMC10355019

[brb371706-bib-0068] Turc, G. , M. Hadziahmetovic , S. Walter , et al. 2022. “Comparison of Mobile Stroke Unit With Usual Care for Acute Ischemic Stroke Management: A Systematic Review and Meta‐analysis.” JAMA neurology 79: 281–290. 35129584 10.1001/jamaneurol.2021.5321 PMC8822443

[brb371706-bib-0069] van den Berg, S. A. , S. M. Uniken Venema , H. Reinink , et al. 2022. “Prehospital Transdermal Glyceryl Trinitrate in Patients With Presumed Acute Stroke (MR ASAP): An Ambulance‐Based, Multicentre, Randomised, Open‐Label, Blinded Endpoint, Phase 3 Trial.” Lancet Neurology 21: 971–981. 36058230 10.1016/S1474-4422(22)00333-7

[brb371706-bib-0070] Wang, H. , Y. Tang , X. Rong , et al. 2014. “Effects of Early Blood Pressure Lowering on Early and Long‐Term Outcomes After Acute Stroke: An Updated Meta‐Analysis.” PLoS ONE 9: e97917. 24853087 10.1371/journal.pone.0097917 PMC4031127

[brb371706-bib-0071] Willmot, M. , A. Ghadami , B. Whysall , W. Clarke , J. Wardlaw , and P. M. Bath . 2006. “Transdermal Glyceryl Trinitrate Lowers Blood Pressure and Maintains Cerebral Blood Flow in Recent Stroke.” Hypertension 47: 1209–1215. 16682611 10.1161/01.HYP.0000223024.02939.1e

[brb371706-bib-0072] Willmot, M. , J. Leonardi‐Bee , and P. M. Bath . 2004. “High Blood Pressure in Acute Stroke and Subsequent Outcome: A Systematic Review.” Hypertension 43: 18–24. 14662649 10.1161/01.HYP.0000105052.65787.35

[brb371706-bib-0073] Wolcott, Z. C. , and S. W. English . 2024. “Artificial Intelligence to Enhance Prehospital Stroke Diagnosis and Triage: A Perspective.” Frontiers in Neurology 15: 1389056. 38756217 10.3389/fneur.2024.1389056 PMC11096539

[brb371706-bib-0074] Yu, K. , Y. Sun , K. Guo , J. Peng , and Y. Jiang . 2023. “Early Blood Pressure Management in Hemorrhagic Stroke: A Meta‐Analysis.” Journal of Neurology 270: 3369–3376. 36884070 10.1007/s00415-023-11654-w

[brb371706-bib-0075] Yuan, F. , F. Yang , J. Zhao , et al. 2021. “Controlling Hypertension After Severe Cerebrovascular Event (CHASE): A Randomized, Multicenter, Controlled Study.” International Journal of Stroke 16: 456–465. 32525464 10.1177/1747493020932784

[brb371706-bib-0076] Yuan, X. , Q. Gan , Y. Zhang , et al. 2025. “Prehospital Blood Pressure Lowering in Patients With Ischemic Stroke: A Systematic Review and Meta‐Analysis of Randomized Controlled Trials.” International Journal of Stroke 20: 289–296. 39460529 10.1177/17474930241298445

[brb371706-bib-0077] Zaki, H. A. , S. A. Lloyd , A. Elmoheen , et al. 2023. “Antihypertensive Interventions in Acute Ischemic Stroke: A Systematic Review and Meta‐Analysis Evaluating Clinical Outcomes Through an Emergency Medicine Paradigm.” Cureus 15: e47729. 38021612 10.7759/cureus.47729 PMC10676241

[brb371706-bib-0078] Zhao, J. L. , Z. Y. Du , Y. R. Sun , et al. 2019. “Intensive Blood Pressure Control Reduces the Risk of Progressive Hemorrhage in Patients With Acute Hypertensive Intracerebral Hemorrhage: A Retrospective Observational Study.” Clinical Neurology and Neurosurgery 180: 1–6. 30870760 10.1016/j.clineuro.2019.02.021

[brb371706-bib-0079] Zhou, S. , and C. Shen . 2022. “Avoiding Definitive Conclusions in Meta‐Analysis of Heterogeneous Studies With Small Sample Sizes.” JAMA Otolaryngology–Head & Neck Surgery 148: 1003–1004. 36136342 10.1001/jamaoto.2022.2847

