# Supplementary material for: Blood Pressure Reduction Time Windows in Stroke: A Systematic Review and Network Meta‐Analysis
Source: Brain Behav. 2026 Aug 24;16(8):e71706. doi: 10.1002/brb3.71706 (PMC13504157; doi:10.1002/brb3.71706)
Supplement: Supplementary file 1 [file BRB3-16-e71706-s011.docx]

**Supplementary Tables**

**Supplementary Table 1. Assignment rule.**

| **Author, Year** | **Original treatment window** | **Assigned node** | **Assignment rule** |
| --- | --- | --- | --- |
| G. Li, 2024 | 2 h | Prehospital | Ulta‐acute (< four hours of onset)/prehospital |
| Saver, 2015 | 2 h |  |  |
| Berg, 2022 | 3 h |  |  |
| Ankolekar, 2013 | 4 h |  |  |
| The RIGHT-2 Investigators, 2019 | 4 h |  |  |
| Adnan I. Qureshi, 2016 | 4.5 h | 6 h | Hyper‐acute (< six hours)/hospital |
| Craig S. Anderson, 2013 | 6 h |  |  |
| ATACH investigators, 2010 | 6 h |  |  |
| Craig S Anderson, 2008 | 6 h |  |  |
| Sebastian Koch, 2008 | 8 h | 24 h | Late hyper-acute (<24 hours) |
| C.Y. Hsu, 1987 | 24 h |  |  |
| David J. Eveson, 2007 | 24 h |  |  |
| Niaz Ahmed, 2000 | 24 h |  |  |
| Rui Dong, 2024 | 24 h |  |  |
| Adnan I. Qureshi, 2005 | 24 h |  |  |
| Kenneth S. Butcher, 2013 | 24 h |  |  |
| Mirza Jusufovic, 2014 | 30 h | 48 h | Acute (< 48 hours) |
| Else Charlotte Sandset, 2011 | 30 h |  |  |
| Joachim Schrader, 2003 | 36 h |  |  |
| John F Potter, 2009 | 36 h |  |  |
| Mi Sun Oh, 2015 | 48 h |  |  |
| Markku Kaste, 1994 | 48 h |  |  |
| Liping Liu, 2023 | 48 h |  |  |
| Jiang He, 2014 | 48 h |  |  |
| Eduardo Martinez-Vila, 1990 | 48 h |  |  |
| Kailash Krishnan, 2016 | 48 h |  |  |
| The ENOS Trial Investigators, 2015 | 48 h |  |  |
| Thompson G Robinson, 2010 | 48 h |  |  |
| Fang Yuan, 2021 | 72 h | 1 week | Sub‐acute (< 168 hours) |
| Mark Willmot, 2006 | 120 h |  |  |
| Philip M.W. Bath, 2001 | 120 h |  |  |

**Note:** This table shows how the original treatment windows reported in each included study were categorized into the five predefined intervention nodes (prehospital, 6 h, 24 h, 48 h, and 1 week) for the network meta-analysis.

**Supplementary Table 2. Egger’ s tests.**

| **Std_effect** | **Coefficient** | **Std. err.** | **t** | p>\|t\| | **95% CI** |
| --- | --- | --- | --- | --- | --- |
| Egger test of mortality in IS patients |  |  |  |  |  |
| Slope | -0.007165 | 0.1120228 | -0.06 | 0.95 | (-0.2537255,0.2393954) |
| Bias | 0.3408588 | 0.4773662 | 0.71 | 0.49 | (-0.7098171,1.391535) |
| Test of H_0_: no small-study effects *P*=0.483 |  |  |  |  |  |
| Egger test of mortality in ICH patients |  |  |  |  |  |
| Slope | -0.2190606 | 0.1231955 | -1.78 | 0.10 | (-0.4902121,0.0520908) |
| Bias | 0.684467 | 0.4882169 | 1.40 | 0.19 | (-0.3900911,1.759025) |
| Test of H_0_: no small-study effects *P*=0.189 |  |  |  |  |  |
| Egger test of mortality in unspecified stroke patients |  |  |  |  |  |
| Slope | 0.0451371 | 0.0832629 | 0.54 | 0.60 | (-0.1381234,0.2283975) |
| Bias | -0.6084737 | 0.4515596 | -1.35 | 0.21 | (-1.60235,0.3854023) |
| Test of H_0_: no small-study effects *P*=0.205 |  |  |  |  |  |

**Abbreviations:** Std_effect = standardized effect, Std. err. = standard error, CI = confidence interval, IS = ischemic stroke, ICH = intracerebral hemorrhage.

**Supplementary Table 3. CINeMA Results of mortality in ischemic stroke patients.**

| **Comparison** | **Within-study bias** | **Reporting bias** | **Indirectness** | **Imprecision** | **Heterogeneity** | **Incoherence** | **Confidence rating** | **Reasons for downgrading** |
| --- | --- | --- | --- | --- | --- | --- | --- | --- |
| 24 hours: Reference | No concerns | Low risk | No concerns | Some concerns | No concerns | Major concerns | Low | “Imprecision”, “Incoherence” |
| 48 hours: Reference | No concerns | Low risk | No concerns | Some concerns | No concerns | Major concerns | Low | “Imprecision”, “Incoherence” |
| Prehospital: Reference | No concerns | Low risk | No concerns | No concerns | Some concerns | Some concerns | Moderate | “Heterogeneity”, “Incoherence” |
| 24 hours: 48 hours | No concerns | Low risk | No concerns | Major concerns | No concerns | Some concerns | Low | “Imprecision”, “Incoherence” |
| 24 hours: Prehospital | No concerns | Low risk | No concerns | Major concerns | No concerns | Some concerns | Low | “Imprecision”, “Incoherence” |
| 48 hours: Prehospital | No concerns | Low risk | No concerns | No concerns | Some concerns | Major concerns | Low | “Heterogeneity”, “Incoherence” |

**Note:** The table presents the confidence ratings for each pairwise comparison assessed using the CINeMA framework. Six domains were evaluated: within-study bias, reporting bias, indirectness, imprecision, heterogeneity, and incoherence. Each domain was rated as "No concerns," "Some concerns," or "Major concerns." The overall confidence rating was determined based on the domain ratings, with the specific reasons for downgrading listed in the final column.

**Supplementary Table 4. CINeMA Results of mortality in intracerebral hemorrhage patients.**

| **Comparison** | **Within-study bias** | **Reporting bias** | **Indirectness** | **Imprecision** | **Heterogeneity** | **Incoherence** | **Confidence rating** | **Reasons for downgrading** |
| --- | --- | --- | --- | --- | --- | --- | --- | --- |
| 24 hours: Reference | Major concerns | Low risk | No concerns | Major concerns | No concerns | Major concerns | Very low | “Within-study bias”, “Imprecision”, “Incoherence” |
| 48 hours: Reference | Major concerns | Low risk | No concerns | Major concerns | No concerns | Major concerns | Very low | “Within-study bias”, “Imprecision”, “Incoherence” |
| 6 hours: Reference | Major concerns | Low risk | No concerns | Major concerns | No concerns | Major concerns | Very low | “Within-study bias”, “Imprecision”, “Incoherence” |
| Prehospital: Reference | No concerns | Low risk | No concerns | Some concerns | No concerns | Some concerns | Moderate | “Imprecision”, “Incoherence” |
| 24 hours: 48 hours | Major concerns | Low risk | No concerns | Major concerns | No concerns | Major concerns | Very low | “Within-study bias”, “Imprecision”, “Incoherence” |
| 24 hours: 6 hours | Major concerns | Low risk | No concerns | Major concerns | No concerns | Major concerns | Very low | “Within-study bias”, “Imprecision”, “Incoherence” |
| 24 hours: Prehospital | No concerns | Low risk | No concerns | Some concerns | No concerns | Major concerns | Low | “Imprecision”, “Incoherence” |
| 48 hours:6 hours | Major concerns | Low risk | No concerns | Major concerns | No concerns | Major concerns | Very low | “Within-study bias”, “Imprecision”, “Incoherence” |
| 48 hours: Prehospital | No concerns | Low risk | No concerns | Some concerns | No concerns | Some concerns | Moderate | “Imprecision”, “Incoherence” |
| 6 hours: Prehospital | No concerns | Low risk | No concerns | Some concerns | No concerns | Some concerns | Moderate | “Imprecision”, “Incoherence” |

**Note:** The table presents the confidence ratings for each pairwise comparison assessed using the CINeMA framework. Six domains were evaluated: within-study bias, reporting bias, indirectness, imprecision, heterogeneity, and incoherence. Each domain was rated as "No concerns," "Some concerns," or "Major concerns." The overall confidence rating was determined based on the domain ratings, with the specific reasons for downgrading listed in the final column.

**Supplementary Table 5. CINeMA Results of mortality in unspecified stroke patients.**

| **Comparison** | **Within-study bias** | **Reporting bias** | **Indirectness** | **Imprecision** | **Heterogeneity** | **Incoherence** | **Confidence rating** | **Reasons for downgrading** |
| --- | --- | --- | --- | --- | --- | --- | --- | --- |
| 48 hours: Reference | No concerns | Low risk | No concerns | Some concerns | No concerns | Major concerns | Low | “Imprecision", “Incoherence" |
| Prehospital: Reference | No concerns | Low risk | No concerns | Major concerns | No concerns | Major concerns | Very low | “Imprecision", “Incoherence" |
| 1 week: Reference | No concerns | Low risk | No concerns | Some concerns | No concerns | Some concerns | Moderate | “Imprecision", “Incoherence" |
| 48 hours: Prehospital | No concerns | Low risk | No concerns | Some concerns | No concerns | Some concerns | Moderate | “Imprecision", “Incoherence" |
| 48 hours: 1 week | No concerns | Low risk | No concerns | Some concerns | No concerns | Some concerns | Moderate | “Imprecision", “Incoherence" |
| 1 week: Prehospital | No concerns | Low risk | No concerns | Some concerns | No concerns | Major concerns | Low | “Imprecision", “Incoherence" |

**Note:** The table presents the confidence ratings for each pairwise comparison assessed using the CINeMA framework. Six domains were evaluated: within-study bias, reporting bias, indirectness, imprecision, heterogeneity, and incoherence. Each domain was rated as "No concerns," "Some concerns," or "Major concerns." The overall confidence rating was determined based on the domain ratings, with the specific reasons for downgrading listed in the final column.

**Supplementary Table 6. Sensitivity analysis of the rank of BPR treatment in ischemic stroke patients on mortality at 90 days.**

| **Treatment** | **SUCRA** | **PrBest** | **MeanRank** |
| --- | --- | --- | --- |
| Reference | 69.3 | 22.4 | 1.9 |
| Prehospital | 20.0 | 0.8 | 3.4 |
| 24 h | 26.2 | 14.8 | 3.2 |
| 48 h | 84.5 | 62.1 | 1.5 |

**Abbreviations:** BPR = blood pressure reduction, SUCRA = surface under the cumulative ranking curve, PrBest = probability of being best.

| **Treatment** | **SUCRA** | **PrBest** | **MeanRank** |
| --- | --- | --- | --- |
| Reference | 44.1 | 3.6 | 3.2 |
| Prehospital | 56.0 | 23.9 | 2.8 |
| 6 h | 53.9 | 20.5 | 2.8 |
| 24 h | 61.1 | 39.4 | 2.6 |
| 48 h | 34.9 | 12.6 | 3.6 |

**Supplementary Table 7. Sensitivity analysis of the rank of BPR treatment in intracerebral hemorrhage patients on mortality at 90 days.**

**Abbreviations:** BPR = blood pressure reduction, SUCRA = surface under the cumulative ranking curve, PrBest = probability of being best.

**Supplementary Table 8. Sensitivity analysis of the rank of BPR treatment in unspecified stroke patients on mortality at 90 days.**

| **Treatment** | **SUCRA** | **PrBest** | **MeanRank** |
| --- | --- | --- | --- |
| Reference | 31.6 | 1.0 | 3.1 |
| Prehospital | 20.0 | 2.0 | 3.4 |
| 48 h | 73.2 | 34.4 | 1.8 |
| 1 week | 75.3 | 62.6 | 1.7 |

**Abbreviations:** BPR = blood pressure reduction, SUCRA = surface under the cumulative ranking curve, PrBest = probability of being best.
