# Supplementary material for: Blood Pressure Reduction Time Windows in Stroke: A Systematic Review and Network Meta‐Analysis
Source: Brain Behav. 2026 Aug 24;16(8):e71706. doi: 10.1002/brb3.71706 (PMC13504157; doi:10.1002/brb3.71706)
Supplement: Supplementary file 2 [file BRB3-16-e71706-s017.docx]

**Supplementary Figures**

**Supplementary Figure 1.** Funnel plots for (A) ischemic stroke patients, (B) intracerebral hemorrhage patients, and (C) unspecified stroke patients. The funnel plot was used to evaluate publication bias by examining the association between effect estimates and their precision. Each point corresponds to a single study, and asymmetry in the scatter of points may be indicative of publication bias. Although visual inspection revealed mild asymmetry, Egger's tests did not indicate statistically significant publication bias (A: *p* = 0.483, B: *p* = 0.189, C: *p* = 0.205).

**Supplementary Figure 2.** Transitivity assessments in terms of distributions for (A) age, (B) course of disease, and (C) sample size across the studies. Scatter plots showing the distribution of continuous baseline variables across included studies. Each point denotes an individual study, color-coded by study cohort.

**Supplementary Figure 3.** Surface under the cumulative ranking curve (SUCRA) for mortality in intracerebral hemorrhage patients across five interventions: (A) reference, (B) prehospital blood pressure reduction (BPR) treatment, (C) 6 h BPR treatment, (D) 24 h BPR treatment, and (E) 48 h BPR treatment. This plot displays the rank probabilities for each intervention, from most effective (rank 1, lowest mortality risk) to least effective (rank 5, highest mortality risk). Bar height represents the probability of achieving each specific rank. The SUCRA value reflects the probability that an intervention ranks among the best treatment, with higher values corresponding to more favorable average rankings.

**Supplementary Figure 4.** Surface under the cumulative ranking curve (SUCRA) for mortality in unspecified stroke patients across four interventions: (A) reference, (B) prehospital blood pressure reduction (BPR) treatment, (C) 48 h BPR treatment, and (D) 1 week BPR treatment. This plot displays the rank probabilities for each intervention, from most effective (rank 1, lowest mortality risk) to least effective (rank 4, highest mortality risk). Bar height represents the probability of achieving each specific rank. The SUCRA value reflects the probability that an intervention ranks among the best treatment, with higher values corresponding to more favorable average rankings.

**Supplementary Figure 5.** The forest plots display the subgroup analysis of ischemic stroke examining the association between 48 h blood pressure reduction treatment and mortality. Effect estimates (odds ratios [ORs] with 95% confidence intervals [CIs]) are presented for each subgroup or individual study. The pooled estimate and its 95% CI are indicated by the diamond at the bottom.

**Supplementary Figure 6.** The forest plots display the subgroup analysis of ischemic stroke examining the association between 24 h blood pressure reduction treatment and mortality. Effect estimates (odds ratios [ORs] with 95% confidence intervals [CIs]) are presented for each subgroup or individual study. The pooled estimate and its 95% CI are indicated by the diamond at the bottom.

**Supplementary Figure 7.** The forest plots display the subgroup analysis of intracerebral hemorrhage examining the association between 24 h blood pressure reduction treatment and mortality. Effect estimates (odds ratios [ORs] with 95% confidence intervals [CIs]) are presented for each subgroup or individual study. The pooled estimate and its 95% CI are indicated by the diamond at the bottom.

**Supplementary Figure 8.** The forest plots display the subgroup analysis of intracerebral hemorrhage examining the association between 48 h blood pressure reduction treatment and mortality. Effect estimates (odds ratios [ORs] with 95% confidence intervals [CIs]) are presented for each subgroup or individual study. The pooled estimate and its 95% CI are indicated by the diamond at the bottom.

**Supplementary Figure 9.** The forest plots display the subgroup analysis of unspecified stroke examining the association between 48 h blood pressure reduction treatment and mortality. Effect estimates (odds ratios [ORs] with 95% confidence intervals [CIs]) are presented for each subgroup or individual study. The pooled estimate and its 95% CI are indicated by the diamond at the bottom.

**Supplementary Figure 10.** The forest plots display the subgroup analysis of unspecified stroke examining the association between 1 week blood pressure reduction treatment and mortality. Effect estimates (odds ratios [ORs] with 95% confidence intervals [CIs]) are presented for each subgroup or individual study. The pooled estimate and its 95% CI are indicated by the diamond at the bottom.

**Supplementary Figures 11.** The forest plots display the subgroup analysis of mortality stratified by different reference types in ischemic stroke patients for (A) prehospital blood pressure reduction (BPR) treatment, (B) 24 h BPR treatment, and (C) 48 h BPR treatment. Effect estimates (odds ratios [ORs] with 95% confidence intervals [CIs]) are presented for each subgroup or individual study. The pooled estimate and its 95% CI are indicated by the diamond at the bottom.

**Supplementary Figures 12.** The forest plots display the subgroup analysis of mortality stratified by different reference types in intracerebral hemorrhage patients for (A) prehospital blood pressure reduction (BPR) treatment, (B) 6 h BPR treatment, (C) 24 h BPR treatment, and (D) 48 h BPR treatment. Effect estimates (odds ratios [ORs] with 95% confidence intervals [CIs]) are presented for each subgroup or individual study. The pooled estimate and its 95% CI are indicated by the diamond at the bottom.

**Supplementary Figures 13.** The forest plots display the subgroup analysis of mortality stratified by different reference types in unspecified stroke patients for (A) prehospital blood pressure reduction (BPR) treatment, (B) 48 h BPR treatment, and (C) 1 week BPR treatment. Effect estimates (odds ratios [ORs] with 95% confidence intervals [CIs]) are presented for each subgroup or individual study. The pooled estimate and its 95% CI are indicated by the diamond at the bottom.

**Supplementary Figure 14.** Network sensitivity analysis of mortality after excluding small-sample studies for (A) ischemic stroke, (B) intracerebral hemorrhage, and (C) unspecified stroke patients. Data are odds ratios (ORs) with 95% confidence intervals (CIs) in a row‑to‑column format. ORs > 1 indicate that the column intervention is associated with a higher risk of mortality than the row intervention. Statistical significance is indicated when the 95% CI does not cross 1.

**Supplementary Figure 15.** Sensitivity analysis of surface under the cumulative ranking curve (SUCRA) for mortality in ischemic stroke patients across four interventions: (A) reference, (B) prehospital blood pressure reduction (BPR) treatment, (C) 24 h BPR treatment, and (D) 48 h BPR treatment. This plot displays the rank probabilities for each intervention, from most effective (rank 1, lowest mortality risk) to least effective (rank 4, highest mortality risk) from a sensitivity analysis that excluded small-sample studies. Bar height represents the probability of achieving each specific rank. The SUCRA value reflects the probability that an intervention ranks among the best treatment, with higher values corresponding to more favorable average rankings.

**Supplementary Figure 16.** Sensitivity analysis of surface under the cumulative ranking curve (SUCRA) for mortality in intracerebral hemorrhage patients across five interventions: (A) reference, (B) prehospital blood pressure reduction (BPR) treatment, (C) 6 h BPR treatment, (D) 24 h BPR treatment, and (E) 48 h BPR treatment. This plot displays the rank probabilities for each intervention, from most effective (rank 1, lowest mortality risk) to least effective (rank 5, highest mortality risk) from a sensitivity analysis that excluded small-sample studies. Bar height represents the probability of achieving each specific rank. The SUCRA value reflects the probability that an intervention ranks among the best treatment, with higher values corresponding to more favorable average rankings.

**Supplementary Figure 17.** Sensitivity analysis of surface under the cumulative ranking curve (SUCRA) for mortality in unspecified stroke patients across four interventions: (A) reference, (B) prehospital blood pressure reduction (BPR) treatment, (C) 48 h BPR treatment, and (D) 1 week BPR treatment. This plot displays the rank probabilities for each intervention, from most effective (rank 1, lowest mortality risk) to least effective (rank 4, highest mortality risk) from a sensitivity analysis that excluded small-sample studies. Bar height represents the probability of achieving each specific rank. The SUCRA value reflects the probability that an intervention ranks among the best treatment, with higher values corresponding to more favorable average rankings.

**Supplementary Figure 18.** Sensitivity analysis was performed by sequentially excluding each drug class to assess the robustness of the findings, and this figure presents the pooled odds ratios (ORs) with 95% confidence intervals (CIs) after the exclusion of each corresponding drug class for mortality in ischemic stroke patients: (A) lisinopril, (B) nimodipine, (C) urapidil, (D) candesartan, (E) prostacyclin (PGI_2_), (F) transdermal glyceryl trinitrate (GTN), (G) valsartan, and (H) combination therapy (two or more antihypertensives).

**Supplementary Figure 19.** Sensitivity analysis was performed by sequentially excluding each drug class to assess the robustness of the findings, and this figure presents the pooled odds ratios (ORs) with 95% confidence intervals (CIs) after the exclusion of each corresponding drug class for mortality in intracerebral hemorrhage patients: (A) candesartan; (B) nimodipine; (C) urapidil; (D) transdermal glyceryl trinitrate (GTN); (E) combination therapy (two or more antihypertensives).

**Supplementary Figure 20.** Sensitivity analysis was performed by sequentially excluding each drug class to assess the robustness of the findings, and this figure presents the pooled odds ratios (ORs) with 95% confidence intervals (CIs) after the exclusion of each corresponding drug class for mortality in unspecified stroke patients: (A) candesartan; (B) urapidil; (C) transdermal glyceryl trinitrate (GTN); (D) magnesium sulfate; (E) combination therapy (two or more antihypertensives).
